# Efficient Expression and Purification of Recombinant Mouse Dimeric IgA

**DOI:** 10.1002/eji.70055

**Published:** 2025-09-15

**Authors:** Antonia Geisse, Tao Zhang, Jonathan Schreiber, Kristina Markova, Sophie Burkhalter, Hedda Wardemann, Andrew J. Macpherson, Tim Rollenske

**Affiliations:** ^1^ Institute of Molecular Medicine and Experimental Immunology University Hospital Bonn Bonn Germany; ^2^ Department of Visceral Surgery and Medicine Inselspital Bern University Hospital University of Bern Bern Switzerland; ^3^ Department for Biomedical Research Visceral Surgery and Medicine University of Bern Bern Switzerland; ^4^ Multidisciplinary Center for Infectious Diseases University of Bern Bern Switzerland; ^5^ German Cancer Research Center B Cell Immunology Heidelberg Germany

**Keywords:** dimeric IgA, immunoglobulin A, J chain, mucosal B cell responses, recombinant monoclonal antibody

## Abstract

Immunoglobulin (Ig) A is the main antibody isotype found on mucosal surfaces in mammals, where it is predominantly present as a dimer. Here we provide an easy, scalable, efficient, and broadly applicable method to produce and purify monoclonal mouse dimeric IgA from single B cell Ig transcripts to study mucosal antibody responses at single‐cell level.

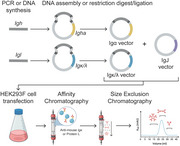

**Abbreviations**
IgA: immunoglobulin A
J chain: joining chain
pIgR: polymeric immunoglobulin receptor
SIgA: secretory IgA

In mammals, large quantities of immunoglobulin A (IgA) antibodies are excreted every day onto the mucosal surfaces. They protect against toxins, viruses and pathogenic bacteria, but can also modulate the composition and the mutualistic behavior of non‐pathogenic members of the microbiota [1]. IgA exists in monomeric and polymeric forms. In serum, monomeric IgA is most prevalent, whereas in mucosal secretions, IgA occurs predominantly as dimers, linked by a single Joining chain (J chain). Dimeric IgA secreted by J chain–expressing short‐lived plasmablasts or long‐lived plasma cells is shuttled across the local epithelium by the polymeric immunoglobulin receptor (pIgR). Upon binding to the pIgR, J chain–containing antibodies are transcytosed to the apical surface of the epithelium, where pIgR is proteolytically cleaved [2]. The cleaved pIgR ectodomain remains bound to dimeric IgA, forming a complex referred to as secretory IgA (SIgA) [3]. SIgA in the intestinal lumen originates from two routes, it is either shuttled across the epithelium lining the gastrointestinal tract or secreted via the biliary excretion pathway [2, 4]. The functional impact of IgA binding in the intestinal lumen depends on the bacterial species and the specific antigen that is recognized [5, 6, 7]. The binding of different IgA antibodies to the same bacterial target can lead to different functional outcomes [5]. Therefore, a better understanding of the role of IgA antibodies in mucosal immunity requires assessments at monoclonal antibody level. Recombinant monoclonal dimeric mouse IgA antibodies have previously been generated and purified using various chromatography strategies [8, 9]. However, a scalable, efficient and broadly applicable method for the production and purification of recombinant mouse dimeric monoclonal IgA has not been described. This is partly due to the limitations of commonly used Protein L‐based purification, which selectively binds only a subset of mouse Igκ light chains encoded by specific *Igkv* genes.

To overcome these limitations, we modified an existing Ig expression vector to produce mouse IgA, generated a J chain expression vector to facilitate the expression of dimeric IgA and tested an *Igkv* gene‐agnostic Igk light chain‐based IgA purification strategy. The mouse Igα expression vector was generated by inserting the *Igha* gene (Ensembl gene ID = ENSMUSG00000095079) of C57BL/6J mice into a vector backbone previously used for the production of recombinant human or mouse IgG (Figure 1A) [10, 11]. The vector was designed to allow for directional insertion of *Igh* genes obtained from single B cells using restriction digestion and ligation or by DNA assembly [12]. To produce dimeric IgA, a eukaryotic expression vector encoding mouse C57BL/6J J chain (Ensembl gene ID = ENSMUSG00000067149) was generated (Figure 1B). To test whether dimeric IgA could be produced, we cloned the Igh and Igk light chains of antibody B8‐030 [5], originating from a mature naïve mouse B cell, into our Igα and Igκ expression vectors and co‐transfected HEK‐293F cells with the corresponding plasmid DNA in a 1:1:1 ratio. After 6 days of culture, the cell supernatants contained on average 31.3 ± 7.9 µg/mL IgA, ranging from 22.1 to 42.2 µg/mL as measured by ELISA (Figure 1C). Gel electrophoresis and subsequent detection of mouse IgA‐containing cell supernatants by immunoblotting identified a single high‐molecular‐weight band under non‐reducing (Figure 1D,E) and a low‐molecular‐weight band under reducing conditions (Figure 1F,G), corresponding to the molecular weight of commercially available dimeric and monomeric IgA, respectively. Immunoblotting confirmed the presence of the J chain in cell supernatants corresponding to the high‐molecular‐weight band (Figure 1H,I), suggesting that dimeric IgA had efficiently formed.

**FIGURE 1:**
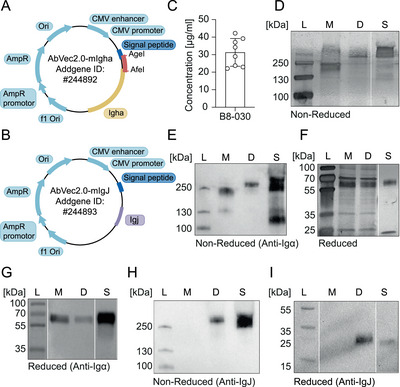
Expression of recombinant mouse dimeric monoclonal IgA. Schematic representation of the eukaryotic expression vector AbVec2.0‐mIgA (A) and AbVec2.0‐mIgJ (B). (C) Concentration of monoclonal mouse negative control IgA antibody in cell culture supernatant determined by ELISA. Silver staining (D, F) and Western Blotting of mouse IgA (E, G) or mouse IgJ (H, I) of B8‐030 mIgA in cell culture supernatant expressed as monomer or dimer under non‐reducing (D, E, H) or reducing (F, G, I) conditions. Ladder (L), monomeric (M), dimeric (D) and commercial Standard (S) IgA fractions are shown. Data are representative of two (D–I) or nine (C) independent experiments.

To purify the dimeric IgA, we used two different strategies. For antibodies encoded by a subset of *Igkv1* [13], *Igkv5* and *Igkv12* [14, 15] variable gene families (Table S1), we used the selective Protein L‐based affinity resin and Glycine‐based elution conditions at pH 3 as previously reported [12]. To facilitate the purification of IgA antibodies independently of their *Igkv* gene usage, we used affinity resin targeting the Igκ light chain and elution using 0.1 M citric acid buffer at pH 2 (Figure 2A–D). To test if these elution conditions had any impact on antigen binding, two mouse monoclonal antibodies against Hen egg lysozyme (HyHEL‐10) and *Plasmodium falciparum* circumsporozoite protein (2A10) were expressed and purified. Antigen binding of the purified HyHEL‐10 and 2A10 antibodies was confirmed by ELISA (Figure 2E). For cleaner preparations, residual monomeric IgA was removed by size exclusion chromatography (Figure 2F).

**FIGURE 2:**
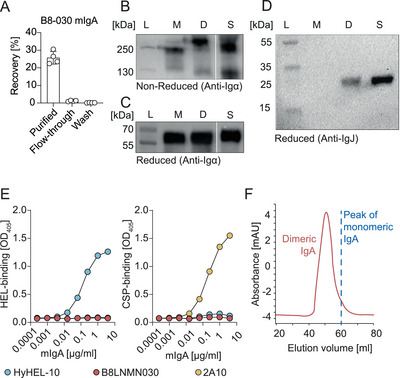
Purification of mouse dimeric monoclonal IgA. (A) Percentage of IgA recovered after affinity purification. Western blot analysis of IgA heavy chain (B,C) or IgJ (D) of purified IgA under non‐reducing (B) and reducing conditions (C,D). (E) Antigen‐binding by ELISA of purified monoclonal IgA antibodies HyHEL‐10 (light blue) and 2A10 (yellow) to Hen egg lysozyme (HEL) (left) and Circumsporozoite protein (CSP) (right), respectively. Negative control IgA antibody B8‐030 is shown in red. (F) Size‐exclusion chromatography of dimeric IgA (red) and the peak corresponding to monomeric IgA are shown (blue dashed line). Ladder (L), monomeric (M), dimeric (D) and commercial standard (S) IgA fractions are shown. Data are representative of two (B–F) or four (A) independent experiments.

In summary, by co‐transfecting HEK‐293F cells with eukaryotic expression vectors for mouse Igα, Igκ (or Igλ) and IgJ chain followed by *Igkv*‐independent affinity purification, we provide an easy‐to‐use, reliable and scalable method for the efficient expression cloning, production and purification of mouse monoclonal dimeric IgA. This method enables the generation of large sets of monoclonal dimeric IgA antibodies to study the antigen targets of mucosal antibody responses at the single‐cell level and test the individual functional in vivo effect of monoclonal IgA antibodies in mucosal immunity.

## 1 Methods

### 1.1 Vectors

Igα, Igκ, Igλ and IgJ eukaryotic expression vector constructs are available and distributed by Addgene (https://www.addgene.org/Hedda_Wardemann/).

### 1.2 Igα Expression Vector Preparation for Cloning

Mouse Igα (Addgene plasmid ID #244892), Igκ (Addgene plasmid # 127157) and Igλ (Addgene ID plasmid # 127156) eukaryotic expression vectors were cut using AgeI‐HF and AfeI, PmlI or MscI restriction enzymes (NEB), respectively, for 2 h at 37°C in Cutsmart buffer (NEB). After Antarctic Phosphatase (NEB) treatment, the vectors were column‐purified using the Gel & PCR Cleanup kit (Macherey & Nagel), both according to the manufacturer's instructions. Re‐ligation efficiency was tested and vector was prepared for ligation or DNA assembly as previously described [12].

### 1.3 Igα Expression Vector Cloning by Restriction Digestion and Ligation

*Igh* and *Igk* or *Igl* transcripts were amplified by PCR using primers with 5′ and 3′ overhangs containing AgeI and AfeI (*Igh* and *Igk*) or MscI (*Igl*) restriction sites, as previously described with sequence adaptations [12]. Mouse Igα, Igκ and Igλ‐specific 5′ and 3′ overhangs can be found in Table S2.

### 1.4 Igα Expression Vector Cloning by DNA Assembly

*Igh* and *Igk* or *Igl* chain transcripts were either amplified by PCR using primer overhangs with 5′ and 3′ ends, which are homologous to the respective expression vectors, or synthesized (Twist Biosciences) to allow direct cloning by DNA assembly into expression vectors as previously described [12]. Mouse Igα, Igκ and Igλ‐specific 5′ and 3′ overhangs can be found in Table S2.

### 1.5 Expression of Recombinant Monoclonal Dimeric Mouse IgA

Recombinant antibody production was performed as previously described [12], adapted for the additional co‐transfection of IgJ expression vector (Addgene plasmid ID #244893). Briefly, PEI‐mediated co‐transfection was performed in HEK293F suspension cells with Igα, corresponding Igκ and IgJ vector in equal amounts at 1.5 µg vector/mL cell culture. After the addition of an equal amount of Excell medium 24 h later, the cells were cultured for an additional 5 days under constant agitation and the supernatant was harvested by centrifugation. Control monoclonal monomeric mouse IgA was generated using the same co‐transfection protocol without the addition of IgJ vector.

### 1.6 Antibody Purification Using Affinity Chromatography

To isolate monoclonal dimeric IgA using gravity flow columns, cell culture supernatants were incubated overnight at 4°C on a rotator with CaptureSelect LC‐kappa affinity matrix (Thermo Fisher Scientific). The required amount of beads were washed with 50 mL PBS by centrifugation for 10 min at 4000 *g* at 4°C. The amount of beads was calculated so that the maximum binding capacity matches the amount of total mouse IgA in the cell culture supernatant. The gravity column was equilibrated with 3 column volumes (CVs) of PBS. After equilibration, the cell culture supernatants were centrifuged at 4000 *g* for 10 min at 4°C, supernatant was removed and beads were transferred to the equilibrated column and emptied by gravity flow. Subsequently, the column was washed with 3 CVs of PBS. To elute the sample, the column was placed in an empty collection tube, and 250 µL 0.1 M citric acid buffer (pH 2.0) was added onto the column, emptied by gravity flow or by applying mild pressure and neutralized with 1:2.5 addition of 1 M Tris‐HCl (pH 9).

Similarly, Protein L‐based purification was performed using Protein L‐coated agarose beads (Pierce) using 225 µL 0.1 M Glycine buffer (pH 3.0) for elution and 1:10 addition of 1 M tris‐HCl (pH 8) for neutralization as previously described [12].

For large cell culture supernatant quantities above 100 mL, recombinant negative control mouse IgA antibody B8‐030 was purified on an Äkta Start system (Cytiva) using a Tricorn 10/20 Column (Cytiva) packed with CaptureSelect LC‐kappa (mouse) affinity matrix (Thermo Fisher Scientific). The column was equilibrated with 5 CVs of PBS, IgA‐containing cell culture supernatant was loaded to the column at a flow rate of 1 mL/min. Subsequently, the column was washed with 10 CVs of PBS. IgA was eluted with 0.1 M citric acid buffer, pH 2 and eluted fractions were neutralized by 1:2.5 addition of 1 M Tris, pH 9.

IgA‐containing fractions were pooled, concentrated using a spin concentrator with a 100 kDa cut‐off membrane (Millipore) and dialyzed (Slide‐A‐Lyzer dialysis devices, Pierce) to 5 L of PBS or 0.9% (w/v) NaCl overnight at 4°C.

### 1.7 Size‐Exclusion Chromatography

Size‐exclusion chromatography was performed on an Äkta Start system (Cytiva) using a HiPrep 16/60 Sephacryl S‐300 HR column (Cytiva) to separate dimeric from monomeric and polymeric IgA. The column was equilibrated with HEPES buffer (20 mM Hepes, 150 mM NaCl, pH 7.4) at a flow rate of 1 mL/min. Fractions corresponding to monomeric and dimeric IgA were collected based on UV absorbance at 280 nm.

### 1.8 Concentration ELISA

IgA concentrations were determined by ELISA as previously described with minor modifications [5]. Briefly, 96‐ or 384‐well high‐binding plates (Costar) were coated with goat anti‐mouse IgA (Southern Biotech) at a concentration of 2 µg/mL in PBS. Plates were washed three times with water before incubation for 1 h with blocking buffer (2 mM EDTA, 0.05 % Tween‐20 in PBS) and washed again.

Purified Mouse IgA k Isotype control (BD Biosciences) was used as standard. Standard and sample dilutions were incubated for 1 h. Plates were washed again with water before incubation with Goat anti‐Mouse IgA (α‐chain specific)—Peroxidase‐conjugated antibody (Sigma) in blocking buffer. Incubation with the secondary antibody was for 1 h and unbound antibodies were removed by washing three times with water. Assays were developed using TMB solution (1 tablet of TMB in 10 mL 0.05 M Phosphate‐Citrate Buffer + 2 µL H_2_O_2_). After 15 min the reaction was stopped with Stopping Solution (2 M H_2_SO_4_). Optical densities (OD) were measured at 450 nm.

### 1.9 Antigen ELISA

Antigen ELISA were performed as previously described [5]. Briefly, HEL and CSP were coated at 10 µg/mL overnight at 4°C. Plates were blocked with 2% BSA (w/v) in PBS pH 7.4. Plates were washed three times with 1x PBS pH 7.4 and incubated with serial dilutions of the indicated antibody concentrations in 0.5% BSA in PBS pH 7.4 for 1.5 h. After three washes with PBS, plates were incubated with horseradish peroxidase‐conjugated anti‐mouse IgA detection antibody, washed and developed using azinoethylbenzothiazoline‐6‐sulfonic acid solution. Optical densities (OD) were measured at 405 nm.

### 1.10 SDS‐PAGE

Purified IgA was analyzed by SDS‐PAGE under reducing or non‐reducing conditions using a 4%–20% precast polyacrylamide gel (Bio‐rad). Antibody fractions were mixed with Laemmli buffer, and for reducing conditions, 2‐mercaptoethanol was added before heating the samples at 98°C for 10 min. A total amount of 0.19–0.33 µg (HEK293F cell supernatant), 0.75–1 µg (purified mouse IgA fractions) or 1.25–2.5 µg commercial mouse IgA (BD) was loaded per lane. Full images of Figures 1D–I and 2B–D are shown in Figure S1A–I.

### 1.11 Silver Staining

Proteins were detected using the Pierce Silver Stain Kit (Thermo Fisher Scientific) following the manufacturer's protocol. Briefly, gels were fixed in 30% ethanol and 10% acetic acid, washed with 10% ethanol and ultrapure water, and then sensitized. After staining with silver solution, bands were developed using an enhancer‐containing developer and the reaction was stopped with 5% acetic acid. All steps were performed at room temperature under constant agitation.

### 1.12 Immunoblot

For western blot, the proteins were transferred using a Trans‐Blot Turbo system (Bio‐Rad) onto a microporous polyvinylidene fluoride (PVDF) membrane (Bio‐rad). After incubation with blocking buffer (3% BSA, 0.1% Tween 20, 0.1% NaN_3_ in PBS) for 1 h at room temperature or 24 h at 4°C, the membranes were incubated with Peroxidase‐conjugated rabbit anti‐mouse IgA or IgJ (both Bio‐rad) in blocking buffer for 1 h. Blots were washed three times with 0.05% Tween 20 in PBS for 5 min. IgA or IgJ were detected by SuperSignal West Atto Ultimate Sensitivity Substrate (Thermo Fisher Scientific).

## Conflicts of Interest

The authors declare no conflict of interest.

## Peer Review

The peer review history for this article is available at https://publons.com/publon/10.1002/eji.70055.

## Supporting information




**Supporting file 1**: eji70055‐sup‐0001‐SuppMat.pdf

## Data Availability

The data that support the findings of this study are available in the Supporting Information of this article.
