# Supplementary material for: Efficient Expression and Purification of Recombinant Mouse Dimeric IgA
Source: Eur J Immunol. 2025 Sep 15;55(9):e70055. doi: 10.1002/eji.70055 (PMC12435132; doi:10.1002/eji.70055)
Supplement: Supplementary file 1 — Supporting file 1: eji70055‐sup‐0001‐SuppMat.pdf [file EJI-55-e70055-s001.pdf]

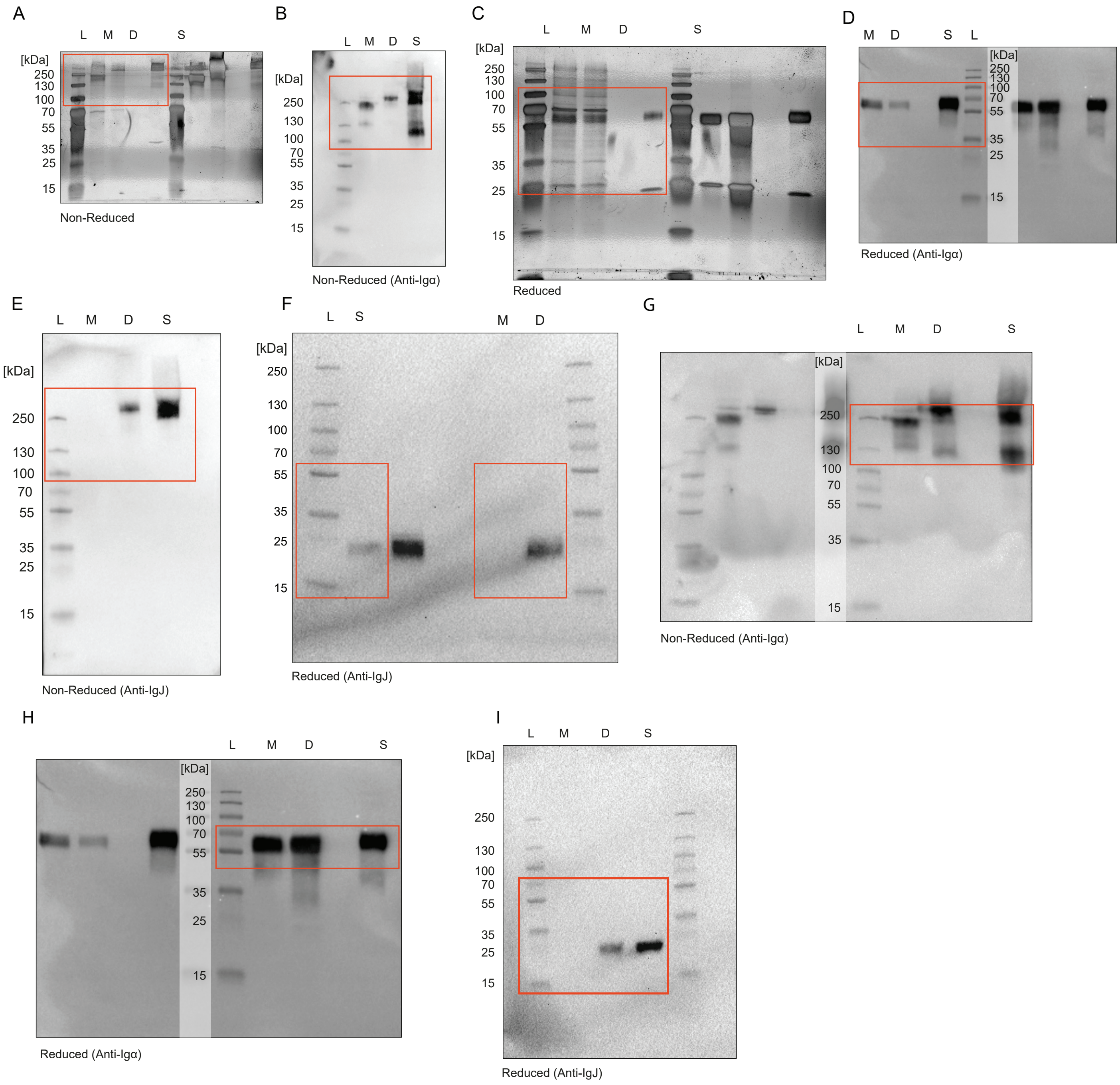

Supplementary Figure 1 Full images of Figures 1D-I and 2B-D showing expression of recombinant mouse monomeric and dimeric IgA. (A, C) Silver staining and (B, D–I) Western blot analysis of B8-030 monoclonal IgA expressed in monomeric (M) or dimeric (D) in cell culture supernatant (A-F) or purified (G-I). Samples were run under non-reducing (A, B, E, G) or reducing (C, D, F, H, I) conditions. Commercial standard mouse IgA (S) was included as a reference. Red boxes indicate the regions shown in the Main Figure.

IGKV family	IGKV gene
IGKV1	IGKV1-88
	IGKV1-99
	IGKV1-110
	IGKV1-117
	IGKV1-117
	IGKV1-122
	IGKV1-132
	IGKV1-133
	IGKV1-135
IGKV5	IGKV5-39 *
	IGKV5-43 *
IGKV12	IGKV12-41

Supporting Information Table 1: Functional mouse IGKV genes reported to be purified by Protein L-based protocols

Asterisk indicates experimentally confirmed light chains of the monoclonal control antibodies B8-030 and HyHEL-10

Insert	5' extension (5' - 3')	3' extension (5' - 3')	Cloning method
IGH	GCAACCGGTGTACATTCC	AGAGCGCTAGA	Restriction digest and ligation
IGK	GCAACCGGTGTACATTCC	**AGC**GCTGTG	Restriction digest and ligation
IGL	GCAACCGGTGTACATTCC	**TG**GCCAGCC	Restriction digest and ligation
IGH	CTAGTAGCAACTGCAACCGGTGTACATTCC	AGAGCGCTAGAAATCCCACC	DNA assembly / synthesis
IGK	CTAGTAGCAACTGCAACCGGTGTACATTCC	GTGCTGATGCTGCACCAACTG	DNA assembly / synthesis
IGL	CTAGTAGCAACTGCAACCGGTGTACATTCC	GCCAGCCCAAGAGCACTCCC	DNA assembly / synthesis

Supporting Information Table 2: 5' and 3' extensions for expression vector cloning

Bold font indicates nucleotides belonging to the *Ig-j* gene; Underlined nucleotides show restriction site
